# Liraglutide Attenuates Atorvastatin-Induced Hepatotoxicity by Restoring GLP-1R Expression and Activating Nrf2 and Autophagy Pathways in Wistar Rats

**DOI:** 10.3390/toxics13070594

**Published:** 2025-07-16

**Authors:** Engy A. Elsiad, Hayat A. Abd El Aal, Hesham A. Salem, Mohammed F. El-Yamany, Mostafa A. Rabie

**Affiliations:** 1 Department of Pharmacology and Toxicology, Faculty of Pharmacy, Cairo University, Cairo 11562, Egypt; hesham.salem@pharma.cu.edu.eg (H.A.S.); mohammed.elyamany@pharma.cu.edu.eg (M.F.E.-Y.); 2 Department of Clinical, Bio-in Molecules, Egyptian Drug Authority (EDA), Cairo 11553, Egypt; drhayatahmed91@gmail.com

**Keywords:** atorvastatin, liraglutide, hepatotoxicity, GLP-1R/Akt, autophagy

## Abstract

HMG-CoA reductase inhibitors, statins, are extensively used to treat hyperlipidemia, coronary artery disease, and other atherosclerotic disorders. However, one of the common side effects of statin therapy is a mild elevation in liver aminotransferases, observed in less than 3% of patients. Atorvastatin and simvastatin, in particular, are most frequently associated with statin-induced liver injury, leading to treatment discontinuation. Recent research has highlighted the antioxidant and anti-inflammatory properties of glucagon-like peptide-1 receptor (GLP-1R) activation in protecting against liver injury. Nonetheless, the potential protective effects of liraglutide (LIRA), a GLP-1R agonist, against atorvastatin (ATO)-induced liver dysfunction have not been fully elucidated. In this context, the present study aimed to investigate the protective role of LIRA in mitigating ATO-induced liver injury in rats, offering new insights into managing statin-associated hepatotoxicity. Indeed, LIRA treatment improved liver function enzymes and attenuated histopathological alterations. LIRA treatment enhanced antioxidant defenses by increasing Nrf2 content and superoxide dismutase (SOD) activity, while reducing NADPH oxidase. Additionally, LIRA suppressed inflammation by downregulating the HMGB1/TLR-4/RAGE axis and inhibiting the protein expression of pY323-MAPK p38 and pS635-NFκB p65 content resulting in decreased proinflammatory cytokines (TNF-α and IL-1β). Furthermore, LIRA upregulated GLP-1R gene expression and promoted autophagic influx via the activation of the pS473-Akt/pS486-AMPK/pS758-ULK1/Beclin-1 signaling cascade, along with inhibiting apoptosis by reducing caspase-3 content. In conclusion, LIRA attenuated ATO-induced oxidative stress and inflammation via activation of the Nrf-2/SOD cascade and inhibition of the HMGB1/TLR-4/RAGE /MAPK p38/NFκB p65 axis. In parallel, LIRA stimulated autophagy via the AMPK/ULK1/Beclin-1 axis and suppressed apoptosis, thus restoring the balance between autophagy and apoptosis.

## 1. Introduction

Cardiovascular disease (CVD) remains one of the leading causes of morbidity and mortality worldwide [1]. For over three decades, statins have been widely prescribed to lower cholesterol levels, thereby reducing CVD-associated risks [2]. Statins exert their lipid-lowering effect by inhibiting the hepatic enzyme HMG-CoA reductase, the rate-limiting step in cholesterol biosynthesis, resulting in a significant reduction in total and LDL cholesterol [3]. While statins are highly effective, concerns have emerged regarding adverse effects, particularly statin-induced liver toxicity (SILT) and muscle toxicity (SIMT) [4]. The mechanisms underlying SILT remain not fully elucidated; however, mitochondrial dysfunction is widely recognized as a central contributor to statin-related hepatotoxicity [5]. Experimental studies, both in vitro and in vivo, have shown that statins induce dose- and time-dependent mitochondrial impairments, including the excessive generation of mitochondrial superoxide and the disruption of mitochondrial membrane potential [6,7,8]. These effects lead to increased oxidative stress, lipid peroxidation, and activation of hepatocellular apoptosis [9,10]. Cerivastatin, for example, was withdrawn from the market due to its severe hepatotoxic profile [11]. In addition to oxidative injury, statins promote immune–inflammatory responses through the oxidative stress-induced activation of danger signaling pathways. Elevated ROS levels can trigger the release of damage-associated molecular patterns (DAMPs) such as high-mobility group box-1 (HMGB1), which in turn activates pattern recognition receptors including toll-like receptors (TLRs) and receptors for advanced glycation end-products (RAGEs) [12]. This activation culminates in the stimulation of the NF-κB pathway and the production of proinflammatory cytokines like TNF-α and IL-1β, thereby perpetuating liver inflammation and injury [13]. Clinically, mild-to-moderate elevations in alanine aminotransferase (ALT) and aspartate aminotransferase (AST) are frequently observed during statin therapy, both in experimental models [14] and in patients [14], with severity often correlating with dose [15]. Hence, regular monitoring of liver enzymes is recommended during statin administration [16]. In parallel, glucagon-like peptide-1 receptor (GLP-1R) agonists such as liraglutide (LIRA) have attracted interest for their extra-pancreatic protective effects. LIRA, a long-acting synthetic GLP-1 analog, is FDA-approved for the treatment of type 2 diabetes mellitus (T2DM) [17]. Beyond glycemic control, GLP-1R is expressed in various tissues including the liver, kidney, heart, and lung [18]. Upon activation, GLP-1R stimulates adenylate cyclase, increases cAMP levels, and activates the PI3K/Akt pathway, which is essential for hepatocyte survival and the regulation of metabolic and inflammatory signals [19,20]. Additionally, GLP-1R activation stimulates the AMPK pathway, a key regulator of autophagy, allowing for the clearance of damaged cellular components and preservation of liver homeostasis [21]. Recent studies have shown that liraglutide exerts antioxidant and anti-inflammatory properties in various hepatic injury models. In NAFLD models, LIRA reduced oxidative stress and inflammatory cytokine levels, including TNF-α and TGF-β1 [22]. It has also been shown to inhibit NF-κB activation in endothelial cells, thereby decreasing IL-1β and TNF-α production [23]. Therefore, the present study aimed to investigate the hepatoprotective mechanisms of liraglutide against atorvastatin-induced liver injury in rats. Specifically, we focused on the modulation of GLP-1R signaling, autophagy-related pathways, and inflammatory mediators such as HMGB1, TLR4, and NF-κB to uncover the therapeutic potential of LIRA in mitigating statin-associated hepatotoxicity, as well as the interplay between autophagy and apoptosis.

### 1.1. Drugs and Chemicals

Liraglutide (LIRA; 1.2 mg injection; Victoza^®^) was obtained from Novo Nordisk, Denmark, whereas Atorvastatin (ATO; 40 mg/tablet; Ator^®^) was acquired from EPICO, Egypt. Unless stated otherwise, all other chemicals used in this study were purchased from Sigma-Aldrich. Furthermore, all reagents were of the highest analytical grade and purity to guarantee the accuracy and reliability of the experimental outcomes.

### 1.2. Animals

#### 1.2.1. Ethical Approval

All experimental procedures and manipulations were performed in accordance with ARRIVE guidelines and the Guide for the Care and Use of Laboratory Animals protocol and approved by the Research Ethics Committee, Faculty of Pharmacy, Cairo University, Cairo, Egypt (Permit number 3021). All efforts were made to minimize animal pain or suffering during experimentation.

#### 1.2.2. Source and Housing Conditions

In the current study, a total of 78 adult male Wistar-albino rats (160–200 g) were obtained from the animal house of the National Organization for Drug Control and Research (NODCAR; Giza; Egypt). The animals were housed under controlled environmental conditions (24 ± 2 °C temperature, 60–70% relative humidity, 12 h light/dark cycle). All rats had free access to a standard diet and water. Animals were allowed to acclimate for one week prior to the initiation of the experiments.

#### 1.2.3. Induction of Liver Injury

The ATO-induced liver dysfunction dose regimen was established based on preliminary study aimed at identifying the optimal dose capable of inducing liver dysfunction within a four-week period. The 28-day duration was selected in accordance with multiple previous studies [1,2], which commonly utilize this time frame for assessing hepatotoxic effects. For the current study, a reference dose of 30 mg/kg was chosen, as it corresponds to the human equivalent dose cited in earlier research [2]. To evaluate the dose-dependent hepatotoxic response of ATO, two lower doses (7.5 and 15 mg/kg) and two higher doses (60 and 120 mg/kg) were included, following a two-fold stepwise decrease and increase from the reference dose.

#### 1.2.4. Experimental Design

##### Preliminary Study: Assessment of ATO-Induced Liver Dysfunction and Dose Selection

A preliminary study was conducted to identify the optimal dose of ATO capable of inducing liver dysfunction. Thirty rats were randomly divided into six groups (*n* = 5 per group). Group 1 received normal saline (p.o.) and served as the control. Groups 2–6 received ATO orally at increasing doses of 7.5, 15, 30, 60, and 120 (mg/kg/day), respectively, for 28 consecutive days.

At the end of the treatment period, blood samples were collected from the retro-orbital plexus to assess liver function markers, specifically ALT and AST. Animals were then euthanized by cervical dislocation under anesthesia. Livers were promptly excised, rinsed with ice-cold saline, and fixed in 10% neutral buffered formalin for histopathological evaluation. Based on the observed changes in liver function tests and histopathological findings, a dose of 60 mg/kg/day was selected as a submaximal dose capable of inducing significant liver injury. This dose was chosen for use in the main experimental phase.

##### Main Study: Evaluation of LIRA’s Protective Effects Against ATO-Induced Liver Dysfunction

Following the preliminary study, the main experiment was designed to investigate the hepatoprotective effects of LIRA against ATO-induced liver dysfunction. Forty-eight rats were randomly divided into eight groups (*n* = 6/group) as follows: Group 1 received normal saline and served as the normal control. Group 2 and 3 received LIRA at a dose range (0.6 and 1.2 mg/kg; i.p.) chosen based on previous preclinical studies on liver that showed liver protection on both doses [3,4], respectively, to act as the normal drug control groups. Group 4–8 received ATO (60 mg/kg; p.o.), where Group 4 was left without treatment to be designed as the ATO group. Group 5 and 6 were treated with LIRA (0.6 mg/kg; i.p.) [4], with Group 5 receiving treatment from day 1 concurrently with ATO administration, while Group 6 started treatment on day 15. Similarly, Group 7 and 8 were treated with a higher dose of LIRA (1.2 mg/kg; i.p.) [4], with Group 7 starting treatment from day 1 alongside ATO and Group 8 beginning treatment on day 15.

All groups received treatment for 28 consecutive days. Atorvastatin (ATO) was administered orally (p.o.), and liraglutide (LIRA) was administered intraperitoneally (i.p.) Twenty-four hours after administering the final dose of ATO or LIRA, blood samples were collected from the retro-orbital sinus under light anesthesia to obtain sera for liver function evaluation (ALT and AST). Subsequently, the rats were euthanized by cervical dislocation under anesthesia, and their livers were excised and rinsed with saline. The left lobe (*n* = 3/group) was preserved in 10% formal saline for histopathological examination using hematoxylin and eosin (H&E) staining.

Based on the results of liver function tests and histopathological evaluations, LIRA at a dose of 1.2 mg/kg administered from day 1 demonstrated the most effective protective effect against ATO-induced liver dysfunction. Therefore, this treatment regimen was selected for further mechanistic investigations to elucidate LIRA’s protective role in mitigating ATO-induced hepatotoxicity.

Afterwards, the right lobe (*n* = 6/group) from the selected groups was divided into three portions for molecular investigations. The 1st portion was homogenized in phosphate-buffered saline to prepare a 10% homogenate for ELISA analysis (Set 1). The 2nd portion was treated with a lysis buffer for RT-PCR analysis (Set 2), and the final portion was submerged in RIPA buffer supplemented with phosphatase and protease inhibitors for Western blot analysis (Set 3).

### 1.3. Biochemical Evaluation

#### 1.3.1. Assessment of Liver Enzymes

Serum aspartate aminotransferase (AST) and alanine aminotransferase (ALT) activities were determined colorimetrically using available commercial kits (Spectrum diagnostics, Cairo, Egypt).

#### 1.3.2. Evaluation of Hepatic S536 NFκB p65, Nrf2, IL-1β, NADPH Oxidase, SOD, Beclin-1, TNF-α, and Caspase-3 Using ELISA Kits

Samples were collected from the retro-orbital plexus sinus under light anesthesia and allowed to clot at room temperature for 30 min, followed by centrifugation at 3000 rpm for 10 min at 4 °C. The serum was then collected and stored at −80 °C until use.

MyBioSource ELISA kits (San Diego, CA, USA) were utilized to assess pS536-NFκB p65 (Cat: #MBS9511033), Nrf2 (Cat:#MBS752046), IL-1β (Cat:#MBS2709618), NADPH oxidase (Cat:# MBS2602768), SOD content (Cat:# MBS036924), Beclin-1 (Cat:#MBS901662), and caspase-3 (Cat:# MBS261814). In parallel, Cusabio ELISA kits (Houston, TX, USA) were used to determine TNF-α (Cat:# CSB-E11987r). Notably, pS536-NFκB p65 and Nrf2 were measured using a nuclear protein extract. All the procedures were performed according to the manufacturer’s instructions and the measured parameters were normalized to the protein content using a Bradford assay [5].

#### 1.3.3. Quantitative Real Time PCR Assessment of HMGB1, TLR-4, RAGE, and GLP-1R

Quantitative RT-PCR was employed to measure the mRNA expression levels of HMGB1, TLR-4, RAGE, and GLP-1R. Total RNA was extracted using the RNeasy Mini Kit (QIAGEN, Germantown, MD, USA), and the purity of the RNA was assessed by spectrophotometry at A260/A280. Equal amounts of RNA were then reverse-transcribed into cDNA using a reverse transcription kit (Applied Biosystems, CA, USA) according to the manufacturer’s instructions. The quantitative RT-PCR was performed with a PCR mixture containing 1 mol/L of each primer and SYBR Green Master Mix (Applied Biosystems, CA, USA) following the manufacturer’s protocol. The primer sequences are listed in Table 1. The amplification conditions were set to 95 °C for 10 min, followed by 40 cycles of denaturation at 95 °C for 15 s and annealing/extension at 60 °C for 10 min. The relative expression of the target genes was calculated using the 2^−ΔΔCT^ method [6], with β-actin serving as the reference gene.

#### 1.3.4. Western Blot Analysis of p-AMPK, p-Akt, p-MAPK p38, and p-ULK-1

Hepatic tissues were homogenized in RIPA lysis buffer supplemented with phosphatase and protease inhibitors for 10 min at 4 °C. Protein concentrations were quantified using the Bradford Protein Assay Kit (Bio BASIC Inc., Markham, ON, Canada). Subsequently, 20 μg of protein was loaded onto and separated by sodium dodecyl sulfate-polyacrylamide gel electrophoresis (SDS-PAGE). The proteins were then transferred to a polyvinylidene difluoride (PVDF) membrane, which was blocked with 5% bovine serum albumin (BSA) and incubated overnight at 4 °C with primary antibodies (Thermo Fisher Scientific, Waltham, MA, USA). The primary antibodies used included pS486-AMPK (1:1000; cat#: PA5-36615), pS473-Akt (1:1000; cat#: PA5-85513), pY323-p38 MAPK (1:1000; cat#: PA5-105007), pS758-ULK1 (1:1000; cat#: PA5-105130), and β-actin (1:10,000; cat#: MA1-140). After washing with Tris-buffered saline-Tween 20, the membranes were incubated with a horseradish peroxidase-conjugated secondary antibody (1:5000) for 90 min at room temperature. Protein band density was determined using the Quantity One system (Bio-Rad, Hercules, CA, USA). Beta-actin was used as the loading control, and the results were normalized to beta-actin levels.

### 1.4. Histopathological Examination

Liver samples (*n* = 3/group) were fixed in 10% formol saline for 24 h. Samples were washed with serial dilutions of alcohol, cleared in xylene, and embedded in paraffin at 56 °C for 24 h. Sections (4 µm) were trimmed by a sledge microtome, stained with hematoxylin and eosin (H&E), and then examined by light microscope in a double-blinded manner.

### 1.5. Statistical Analysis

Normality and homogeneity of variances were assessed using the Shapiro–Wilk test and the Brown–Forsythe test, respectively, to ensure the suitability of parametric analysis. Statistical comparisons were conducted using one-way ANOVA, followed by Tukey’s post hoc test for multiple comparisons. Data are presented as mean ± standard deviation (SD). Statistical analysis and graphical representations were performed using GraphPad Prism^®^ version 9 (GraphPad Software Inc., San Diego, CA, USA). A *p*-value of <0.05 was considered statistically significant.

## 2. Results

### 2.1. Preliminary Study: Assessment of ATO-Induced Liver Dysfunction and Dose Selection

#### 2.1.1. Effect of ATO Dose Regimens (7.5, 15, 30, 60, and 120 mg/kg) on Liver Function Tests

**Figure 1:**
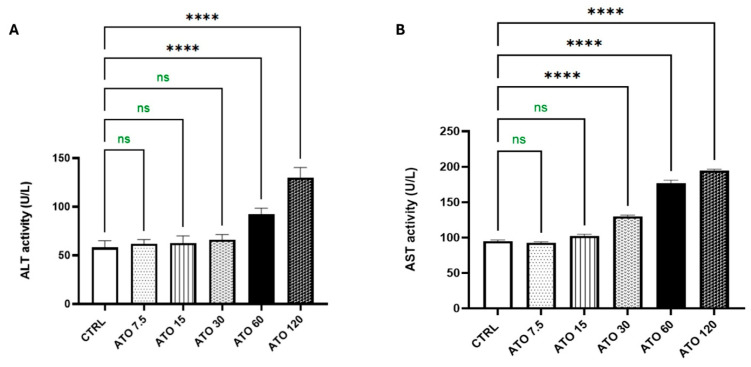
Effect of ATO dose regimens (7.5, 15, 30, 60, and 120 mg/kg) on the hepatic (**A**) ALT and (**B**) AST activities in rat model. Data are expressed as mean (5/group) ± S.D., analyzed using one-way ANOVA followed by Tukey’s post hoc test. **** *p* < 0.0001. CTRL: control; ATO: atorvastatin; ALT: alanine aminotransferase; AST: aspartate aminotransferase. “ns” indicates non-significant differences.

Compared to the CTRL group, the administration of ATO for 28 consecutive days did not result in any significant elevation in liver enzymes at doses of 7.5 and 15 mg/kg, as both ALT and AST levels remained nearly within normal ranges. At a dose of 30 mg/kg, ALT levels remained unaffected; however, a significant increase of 37% in AST activity was observed compared to the CTRL group. Higher doses of ATO, specifically 60 and 120 mg/kg, led to marked elevations in both ALT and AST levels. At 60 mg/kg, ALT and AST increased by 60% and 86%, respectively, while at 120 mg/kg, ALT and AST showed increases of about 1.24-fold and 1-fold, respectively, relative to the CTRL group (Figure 1). Based on these findings, liver tissues from the ATO-treated groups (30, 60, and 120 mg/kg) were subjected to H&E staining for histopathological evaluation.

#### 2.1.2. Effect of ATO Dose Regimens (30, 60, and 120 mg/kg) on Histopathological Investigation

**Figure 2:**
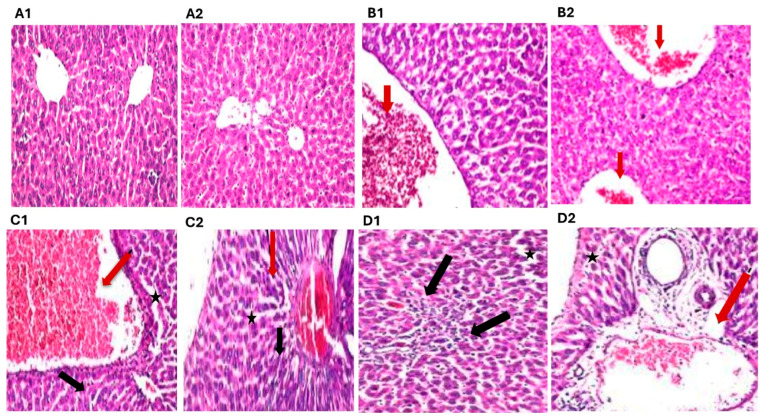
Effect of ATO dose regimens (30, 60, and 120 mg/kg) on hepatic histopathology in rat model. Photomicrographs representing H&E-stained hepatic tissues of (**A**) CTRL group, (**B**) ATO (30 mg/kg) group, (**C**) ATO (60 mg/kg) group, and (**D**) ATO (120 mg/kg) group (scale bar = 25 µm). Black arrow: necrotic changes in hepatocytes; star: sinusoidal dilatation; red arrow: high number of mononuclear inflammatory cells CTRL: control; ATO: atorvastatin.

Histological analysis of liver sections stained with H&E from the CTRL group revealed a normal hepatic architecture, with intact pericentral hepatocytes and a well-preserved central vein structure (Figure 2(A1,A2)). However, liver sections from the group treated with 30 mg/kg of ATO displayed mild pathological changes, including slight dilatation and infiltration of inflammatory cells within the portal area. Notably, there were no signs of fibrosis or necrosis observed in this group (Figure 2(B1,B2)). More pronounced alterations were evident in the livers of rats treated with 60 mg/kg/day of ATO, which showed clear signs of hepatotoxicity. These changes included congestion in the portal area, severe dilation and congestion of the central vein, focal preductal fibrosis, and infiltration of Kupffer cells between hepatocytes, along with the presence of inflammatory cells in the portal region (Figure 2(C1,C2)). Rats receiving the highest dose of ATO (120 mg/kg/day) exhibited marked hepatic damage characterized by extensive dilation and congestion in both portal and central veins, focal necrosis within the parenchyma, and a more prominent infiltration of Kupffer cells (Figure 2(D1,D2)).

Based on the histopathological findings in conjunction with the liver function test results, the ATO dose of 60 mg/kg/day was identified as a submaximal dose sufficient to induce notable liver toxicity over a 28-day treatment period. Thus, this dose was selected for implementation in the main experiment.

### 2.2. Main Study: Evaluation of LIRA’s Protective Effects Against ATO-Induced Liver Dysfunction

#### 2.2.1. Effect of LIRA (0.6 and 1.2 mg/kg) on ALT and AST Levels in ATO-Induced Liver Dysfunction

As compared to the CTRL, administration of ATO for 28 days deteriorated liver function as witnessed by marked increases in ALT and AST activities [F_(7, 40)_ = 38.74 and 42.72, respectively] by 72% and 82%, respectively. However, co-treatment with LIRA at doses of 0.6 and 1.2 mg/kg starting from day 1 to day 28 significantly reduced the ATO-induced elevation of ALT levels by 24 and 45% and AST levels by 15% and 41%, respectively, as compared to the ATO group. Alternatively, starting LIRA administration on day 15 with the same doses (0.6 and 1.2 mg/kg) improved liver function, reducing ALT activities by 18% and 22% and AST activities by 13% and 28%, respectively, as compared to the ATO group.

**Figure 3:**
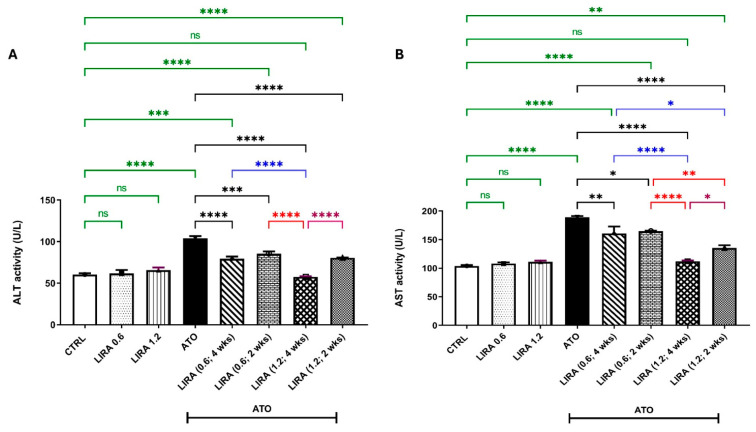
Effect of LD and HD LIRA on the hepatic (**A**) ALT and (**B**) AST activities in ATO-induced liver dysfunction in rat model. Data are expressed as mean (6/group) ± S.D., analyzed using one-way ANOVA followed by Tukey’s post hoc test. * *p* < 0.05; ** *p* < 0.01; *** *p* < 0.001; **** *p* < 0.0001. CTRL: control; LD: low dose; HD: high dose; LIRA: liraglutide; ATO: atorvastatin; ALT: alanine aminotransferase; AST: aspartate aminotransferase. “ns” indicates non-significant differences.

Notably, the concurrent administration of LIRA at a dose of 1.2 mg/kg from day 1 demonstrated the highest level of liver protection, effectively normalizing ALT and AST levels. Although the other LIRA regimen still improved liver function, its protective effects were less pronounced than the early administration of LIRA at a dose of 1.2 mg/kg from day 1 (Figure 3).

#### 2.2.2. Effect of LIRA (0.6 and 1.2 mg/kg) on Histopathological Investigation in ATO-Induced Liver Dysfunction

**Figure 4:**
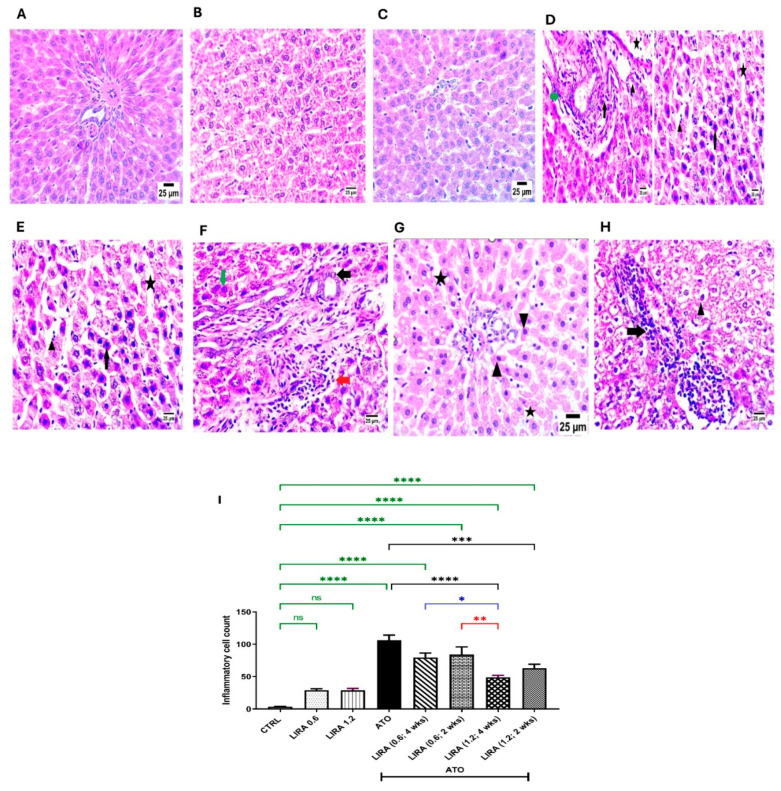
Effect of LD and HD LIRA on the hepatic histopathology in ATO-induced liver dysfunction in rat model. Photomicrographs representing H&E-stained hepatic tissues of (**A**) CTRL group, (**B**) LD LIRA (0.6 mg/kg) group, (**C**) HD LIRA (1.2 mg/kg) group, (**D**) ATO group, (**E**) ATO + LD LIRA group started from day 1, (**F**) ATO + LD LIRA group started from day 15, (**G**) ATO + HD LIRA group started from day 1, and (**H**) ATO + HD LIRA group started from day 15 (scale bar = 25 µm). Panel (**I**) demonstrates inflammatory cell count score. Black arrow: necrotic changes in hepatocytes; star: sinusoidal dilatation; green arrow: portal fibrosis; arrowhead: occasional nuclear pyknosis in some hepatocytes; red arrow: high number of mononuclear inflammatory cells. * *p* < 0.05; ** *p* < 0.01; *** *p* < 0.001; **** *p* < 0.0001. “ns” indicates non-significant differences. CTRL: control; LD: low dose; HD: high dose; LIRA: liraglutide; ATO: atorvastatin.

Examination of liver sections stained with H&E from the CTRL and LIRA-alone groups (0.6 and 1.2 mg/kg) revealed normal histological architectures of the central vein and intact pericentral hepatocytes (Figure 4A–C). Conversely, ATO administration resulted in necrobiotic alterations in hepatocytes, characterized by nuclear pyknosis and acidophilic cytoplasm (arrowhead), along with sinusoidal dilatation (star). Furthermore, ATO triggered portal fibrosis (green arrow), infiltration of mononuclear inflammatory cells (black arrow), and congestion of portal blood vessels (star) with the formation of new bile ductules (arrowhead) (Figure 4D). In contrast, the administration of LD LIRA (0.6 mg/kg) starting on either day 1 or day 15 showed no significant differences compared to the ATO group (Figure 4E,F). However, the concurrent administration of HD LIRA (1.2 mg/kg) from day 1 resulted in mild histopathological alterations, including slight sinusoidal dilatation (star) and occasional nuclear pyknosis in some hepatocytes (arrowhead) (Figure 4G). Notably, administration of HD LIRA (1.2 mg/kg) from day 15 led to more pronounced sinusoidal dilatation with blood engorgement, increased mononuclear inflammatory cell infiltration (arrow), and severe vacuolar degeneration in hepatocytes accompanied by pyknotic nuclei (arrowhead) (Figure 4H). Moreover, the assessment of inflammatory cell count is presented in Figure 4I.

In dose–response screening, the protective effects of LIRA at doses of 0.6 and 1.2 mg/kg were evaluated using liver function markers (ALT and AST) and histopathological examination. The findings indicated that the higher dose of LIRA (1.2 mg/kg), administered alongside ATO from day 1, yielded the most beneficial results. As a result, additional biochemical analyses were performed to further explore the protective mechanisms of LIRA against ATO-induced hepatotoxicity.

### 2.3. Effect of HD LIRA (1.2 mg/kg) on Biochemical Parameters

Group 2 (LIRA) showed no significant difference from group 1 (CTRL) in all parameters assessed; thus, comparisons referred to group 1 only.

#### 2.3.1. HD LIRA Mitigated ATO-Induced Oxidative Stress in Liver

**Figure 5:**
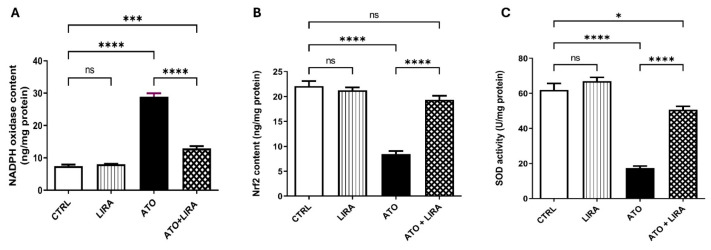
Effect of HD LIRA on the hepatic (**A**) NADPH oxidase, (**B**) Nrf2 content, and (**C**) SOD activity in ATO-induced liver dysfunction in rat model. Data are expressed as mean (6/group) ± S.D., analyzed using one-way ANOVA followed by Tukey’s post hoc test. * *p* < 0.05; *** *p* < 0.001; **** *p* < 0.0001. “ns” indicates non-significant differences. CTRL: control; LIRA: liraglutide; ATO: atorvastatin; NADPH-oxidase: nicotinamide adenine dinucleotide phosphate oxidase; Nrf2: nuclear factor erythroid 2-related factor; SOD: super oxide dismutase.

The ameliorative effect of LIRA against ATO-induced oxidative stress was investigated via determination of NADPH oxidase, Nrf-2, and SOD. Indeed, rats that received ATO showed a significant escalation in hepatic NADPH oxidase content [F_(3, 20)_ = 206.8], by 2.9-fold, together with a decline in Nrf2 content and SOD activity [F_(3, 20)_ = 69.39 and 84.62, respectively] by 62 and 72%, respectively, as compared to the CTRL group (*p* < 0.0001). In contrast, treatment with LIRA (1.2 mg/kg), started from day 1, decreased NADPH oxidase content by 55% and increased Nrf2 content and SOD activity by 1.3- and 1.9-fold, respectively, as compared to the CTRL group (Figure 5).

#### 2.3.2. HD LIRA Ameliorated ATO-Induced Inflammation in Liver

**Figure 6:**
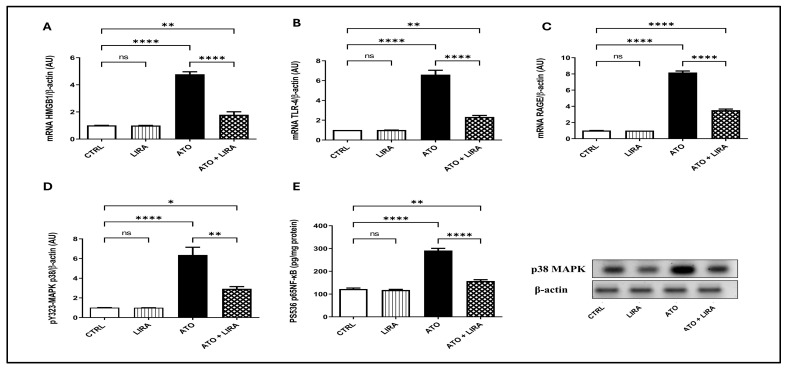
Effect of HD LIRA on the hepatic gene expressions of (**A**) HMGB1, (**B**) TLR-4, and (**C**) RAGE as well as (**D**) pY323-MAPK p38 protein expression and (**E**) pS536-p65NF-kB content in ATO-induced liver dysfunction in rat model. Data are expressed as mean (3–6/group) ± S.D., analyzed using one-way ANOVA followed by Tukey’s post hoc test. * *p* < 0.05; ** *p* < 0.01; **** *p* < 0.0001. “ns” indicates non-significant differences. CTRL: control; LIRA: liraglutide; ATO: atorvastatin; HMGB-1: high-mobility group box-1; TLR-4: toll-like receptor; RAGE: receptor of advanced glycated end-products; p38-MAPK: phosphorylated mitogen-activated protein kinase; p38 NF-kB: phosphorylated nuclear factor kapa-B.

**Figure 7:**
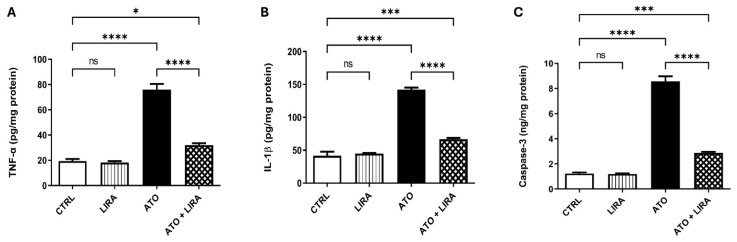
Effect of HD LIRA on the hepatic (**A**) TNFα, (**B**) IL-1β, and (**C**) caspase-3 contents in ATO-induced liver dysfunction in rat model. Data are expressed as mean (6/group) ± S.D., analyzed using one-way ANOVA followed by Tukey’s post hoc test. * *p* < 0.05; *** *p* < 0.001; **** *p* < 0.0001; “ns” indicates non-significant differences. CTRL: control; LIRA: liraglutide; ATO: atorvastatin; TNFα: tumor necrosis factor alpha; IL-1β: Interleukin1β.

To explore the mechanism underlying the anti-inflammatory effect of LIRA against ATO-induced liver damage, the signal transduction of the HMGB-1/RAGE/TLR-4/pY323-MAPK p38/pS536 NF-κB p65 pathway was examined. Notably, administration of ATO upregulated the mRNA expression of HMGB-1, RAGE, and TLR-4 [F_(3, 20)_ = 158.5, 876.6, and 127.7, respectively] by 3.8-, 5.6-, and 7.7-fold, respectively, along with an increase in the protein expression of pY323-MAPK p38 [F_(3, 8)_ = 38.6] by 5.2-fold and p-NF-κB p65 content [F_(3, 20)_ = 181.2] by 1.4-fold, relative to the CTRL group (*p* < 0.0001) (Figure 6). Furthermore, ATO administration increased the downstream proinflammatory cytokines TNFα and IL1β content [F_(3, 20)_ = 106.7 and 165.6, respectively] by 2.9- and 2.4-fold, respectively, as well as apoptotic biomarker caspase-3 [F_(3, 20)_ = 277.0] by 5.9-fold, as compared to the CTRL group (*p* < 0.0001) (Figure 7). On the other hand, LIRA treatment (1.2 mg/kg) succeeded in suppressing inflammatory status and downregulating the gene expression of HMGB-1 (38%), RAGE (43%), and TLR-4 (35%), as well as causing a reduction in the protein expression of p38 MAPK (43%) and the protein contents of p-NF-κB p65 (54%), TNFα (42%), IL1β (47%), and caspase-3 (33%), as compared to the ATO + LIRA group.

#### 2.3.3. HD LIRA Restored the Imbalance Between Autophagy and Apoptosis in ATO-Induced Liver Injury

**Figure 8:**
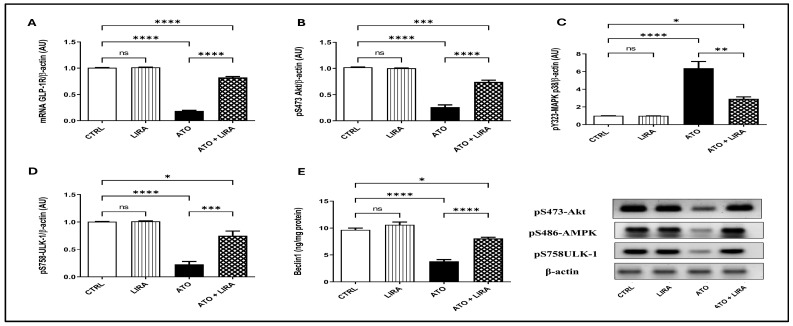
Effect of HD LIRA on the hepatic (**A**) mRNA GLP-1R, as well as the protein expression of (**B**) pS473-Akt, (**C**) pS486-AMPK, and (**D**) pS758-ULK-1 and (**E**) Beclin-1 content in ATO-induced liver dysfunction in rat model. Data are expressed as mean (3–6/group) ± S.D., analyzed using one-way ANOVA followed by Tukey’s post hoc test. * *p* < 0.05; ** *p* < 0.01; *** *p* < 0.001; **** *p* < 0.0001. “ns” indicates non-significant differences. CTRL: control; LIRA: liraglutide; ATO: atorvastatin; GLP-1: glucagon-like peptide-1 receptor; Akt: protein kinase B; AMPK: AMP-activated protein kinase.

Rats that received ATO showed a decrease in the gene expression of GLP-1R [F_(3, 20)_ = 1476] by 81%, in the protein expression of pS473-Akt, pS486-AMPK, and pS758-ULK-1 [F_(3, 8)_ = 169.9, 81.17 and 53.78, respectively] by 75, 77, and 80%, respectively, and in Beclin1 content [F_(3, 20)_ = 74.55] by 60% in comparison to the CTRL group (*p* < 0.0001). In opposition, treatment with LIRA (1.2 mg/kg) stimulated autophagy as demonstrated by an upsurge in GLP-1R gene expression by 3.4-fold together with an increase in the protein expression of p-Akt, p-AMPK, and p-ULK-1 by 1.8-, 2.4-, and 2.5-fold, respectively, in addition to boosting Beclin1 content by 1.1-fold, as compared to the CTRL group (Figure 8).

## 3. Discussion

To the authors’ knowledge, this study is the first to identify and characterize the role of GLP-1R in mitigating ATO-induced liver dysfunction. Indeed, LIRA alleviated oxidative stress via reducing NADPH oxidase and enhancing Nrf2 and SOD levels. Additionally, LIRA downregulated HMGB1, TLR4, and RAGE gene expression, leading to a reduction in MAPK p38 and the inflammatory transcription factor, NF-κB p65, and the associated cytokines (TNF-α and IL-1β), thus suppressing the inflammatory response. Furthermore, LIRA stimulated the GLP-1R/Akt axis, which promoted autophagy through the AMPK/ULK1/Beclin1 pathway, aiding to restore the balance between autophagy and apoptosis.

In the current study, a preliminary experiment was conducted to determine the optimal dose of ATO that induces liver dysfunction within 28 days, a duration commonly used in similar research [1,2]. A reference dose of 30 mg/kg, based on the human equivalent dose from prior studies [2], was tested along with two lower doses (7.5 and 15 mg/kg) and two higher doses (60 and 120 mg/kg), using a two-fold stepwise approach. Among these, 60 mg/kg/day was identified as a submaximal dose that caused significant liver damage based on liver function tests and histopathological analysis and was selected for the main experimental phase.

Following the preliminary study, the main experiment was carried out to evaluate the hepatoprotective effects of LIRA against ATO-induced liver injury. Dose-screening was performed based on prior studies, which utilized 0.6 and 1.2 mg/kg (i.p.) of LIRA to counteract methotrexate-induced hepatotoxicity in rats [4]. Indeed, LIRA (1.2 mg/kg) administered from day 1 demonstrated the most pronounced hepatoprotective effect, as evidenced by liver function tests and histopathological analysis. This effect is likely due to the early administration of LIRA, which may help prevent the onset of irreversible liver necrosis [7] associated with ATO toxicity [8], a process that could begin before noticeable structural damage or enzyme elevation occurs. Consequently, the 1.2 mg/kg dose was chosen for subsequent mechanistic investigations to further elucidate LIRA’s protective role against ATO-induced liver damage.

Atorvastatin (ATO), the most widely used statin, is primarily used to lower blood cholesterol and inhibits the HMG-CoA reductase enzyme in the liver, the rate-limiting step of cholesterol biosynthesis [9]. This inhibition leads to decreased cholesterol production and increased removal of LDL cholesterol from the bloodstream, ultimately lowering overall cholesterol levels and potentially preventing cardiovascular diseases [10]. Notably, long-term use of statins not only lowers cholesterol synthesis but also inhibits the production of isoprenoids [11], which are crucial for synthesizing coenzymes such as coenzyme Q10 (CoQ10). The latter plays vital roles in mitochondrial electron transport and antioxidant defenses [12]. This interference creates an oxidative stress status, impacting cellular mitochondrial function, as observed herein. Indeed, ATO increased NADPH oxidase, generating superoxide anions and subsequent reactive oxygen species (ROS) production [13], and decreased nuclear factor erythroid 2-related factor 2 (Nrf2), a key transcription factor that regulates antioxidant defense mechanisms such as superoxide dismutase (SOD) [14]. In the same line, ATO induced mitochondrial dysfunction and cell apoptosis in HepG2 cells via inhibition of the Nrf2 pathway and a reduction in SOD expression [15]. Indeed, the oxidative stress status created triggers the release of damage-associated molecular pattern (DAMPs), which in turn provokes an inflammatory response and exacerbates tissue damage effects [16]. High-mobility group box 1 (HMGB1), one of the principal DAMPs, is typically located in the nucleus under normal conditions [17]. However, in response to cellular stress or injury, it relocates to the cytoplasm and is subsequently released into the extracellular space. Once in the extracellular space, HMGB1 can engage with pattern recognition receptors such as toll-like receptor (TLR)-4 and the receptor for advanced glycation end-products (RAGE) [18]. These receptors play crucial roles in regulating immune and inflammatory response [19]. Upon activation, TLR4 and RAGE recruit MyD88, an adaptor protein, initiating downstream signaling cascades. This process includes the activation of MAPK p38 and NF-κB p65, leading to the transcription and translation of proinflammatory cytokines such as TNF-α and IL-1β [20,21]. In the current study, ATO upregulated the gene expression of HMGB1, which subsequently promoted the transcription of TLR4 and RAGE. The binding of HMGB1 to TLR4 and RAGE induced the phosphorylation and activation MAPK p38 and NF-κB p65, resulting in increased proinflammatory cytokine production such as TNF-α and IL-1β. Notably, oxidative stress and inflammation status can induce caspase-3 activation, which plays a crucial role in triggering apoptosis [22]. Increased levels of caspase-3 observed herein could be attributed, at least in part, to oxidative stress and inflammatory responses induced by ATO. In parallel, reduced isoprenoids levels trigger a cascade of mitochondrial-mediated apoptotic signaling pathways [23].

In opposition, treatment with LIRA, a GLP-1R agonist, alleviated oxidative stress through several molecular pathways as observed herein and previously [24]. Indeed, activation of GLP-1R triggers the phosphorylation and activation of protein kinase A (PKA), which inhibits NADPH oxidase activity by preventing the translocation of its cytosolic subunits to the membrane, thus reducing ROS production [25]. Another crucial pathway involves activating Nrf2 via the PI3K/Akt signaling cascade. Upon GLP-1R activation, the PI3K/Akt pathway is stimulated, leading to the phosphorylation and activation of Akt, which in turn promotes the nuclear translocation of Nrf2 to enhance the transcription of antioxidant enzymes. By enhancing the expression of these enzymes, Nrf2 activation helps to detoxify ROS and mitigate oxidative stress. This dual action of LIRA observed in the current study, reducing ROS production through NADPH oxidase inhibition and enhancing antioxidant defense via Nrf2 activation and boosting SOD activity, highlights its potential therapeutic efficacy against ATO-induced oxidative stress. In the same context, LIRA mitigated hepatic oxidative stress in diabetic mice via enhancing SOD and GPx activities [26]. Moreover, LIRA inhibited oxidative stress in diet-induced non-alcoholic fatty liver disease (NAFLD) via increasing SOD activity and reducing malondialdehyde (MDA) levels [3]. Furthermore, LIRA attenuated oxidative stress in CCL4-induced acute hepatic injury via stimulating the Nrf2/HO-1 signaling pathway [27]. Notably, mitigation of oxidative stress status reduced HMGB1 release from damaged cells, thereby limiting its ability to bind to TLR4 and RAGE. This reduction in HMGB1 levels consequently decreased the activation of the MAPK p38 and NF-κB p65 pathways, resulting in lower production of proinflammatory cytokines such as TNF-α and IL-1β, and ultimately suppressing inflammation. Additionally, the activation of Akt through GLP-1R stimulation inhibited NF-κB activation by phosphorylating and inactivating IκB kinase (IKK). This prevented the degradation of IκB, thereby keeping NF-κB sequestered in the cytoplasm [28]. Therefore, by activating the PI3K/Akt pathway, the GLP-1R agonist LIRA not only inhibits inflammatory signaling mediated by HMGB1/TLR4/RAGE interactions but also enhances cellular defenses against oxidative stress. In the same line, El Tabaa et al. [29] demonstrated that LIRA displayed hepatoprotective activity against valproic acid (VPA)-induced hepatotoxicity via reducing oxidative stress and inflammation. Indeed, LIRA reduced HMGB1, RAGE, and MDA while increasing SOD and GSH activities compared to the VPA hepatotoxic group. Additionally, LIRA treatment inhibited ischemia-induced nucleocytoplasmic translocation and the release of HMGB1, resulting in reduced levels of TNF-α, IL-1β, IL-6, TLR-4, and RAGE mRNA [30]. Notably, the decrease in oxidative stress and inflammation observed with LIRA treatment could be a reason for the inhibition of apoptosis as evidenced by the reduced levels of caspase-3. Furthermore, GLP-1R activation increases the expression of anti-apoptotic proteins like Bcl-2 and decreases the expression of pro-apoptotic proteins like Bax, leading to a reduction in mitochondrial-mediated apoptosis [31]. Indeed, Yu et al. [26] reported that LIRA decreased the expression of proapoptotic proteins such as caspase-3 and Bax in diabetic rats.

In addition to its antioxidant and anti-inflammatory properties, the GLP-1R agonist LIRA stimulates autophagy, a critical cellular process for degrading and recycling damaged organelles and proteins, thus maintaining cellular homeostasis [32]. Indeed, GLP-1R activation increases intracellular cAMP levels, which subsequently activates PKA, leading to the phosphorylation/activation of AMPK [33]. Activated AMPK promotes autophagy by inhibiting the mammalian target of the rapamycin (mTOR) pathway, a negative regulator of autophagy. This inhibition lifts the suppression of autophagy-related genes, facilitating the autophagic process [34]. Furthermore, AMPK activation phosphorylates Unc-51-like kinase 1 (ULK1), a key initiator of autophagy [35]. Phosphorylated ULK1 interacts with Beclin1, an essential component of the autophagy initiation complex. Beclin1, in collaboration with other autophagy-related proteins, aids in the formation of the autophagosome, the cellular structure responsible for sequestering and degrading damaged organelles and proteins. In the current study, LIRA increased the protein expression of p-AMPK and p-ULK1 as well as Beclin1 content to stimulate autophagic flux, effects that were in accordance with a previous study [36]. Moreover, LIRA enhanced autophagy via stimulation of AMPK signaling pathway to ameliorate NAFLD in diabetic mice [37]. In parallel, activated AMPK lowers intracellular ROS level by increasing the expression of antioxidant defense enzymes [38] and enhances mitochondrial biogenesis via proliferator-activated receptor-γ coactivator-1α [39]. Thus, stimulation of the AMPK signaling cascade not only promotes autophagy but also reduces ATO-induced oxidative stress as witnessed herein. In the same context, a prior study [40] showed that resveratrol-induced AMPK activation diminished ROS production and boosted the activity of the Akt-nNOS pathway. This effect was mediated through the downregulation of NADPH oxidase and the upregulation of SOD activity in hypertensive rats, providing protection against oxidative stress-induced hypertension.

Indeed, a total of nine cohort studies, selected from 535 identified articles and involving 579,256 patients with type 2 diabetes mellitus (T2DM), reported that the use of GLP-1R agonists was associated with a decreased risk of developing hepatocellular carcinoma and experiencing cirrhosis decompensation. These findings underscore the hepatoprotective effects of GLP-1R agonists in T2DM patients and are consistent with previous evidence suggesting their superior hepatoprotective benefits compared to other antidiabetic therapies [41]. Furthermore, evidence from both preclinical and clinical research indicates that injectable GLP-1R agonists positively impact endothelial and heart health by lowering blood pressure, reducing weight, and decreasing levels of inflammatory markers [42]. Increased GLP-1 levels have been shown to reduce serum and liver triglycerides in both normal animal models and in those with high-fat diet-induced insulin resistance [43]. Additionally, combining GLP-1R agonists with sodium-glucose cotransporter 2 (SGLT-2) inhibitors may provide more significant improvements in cardiovascular health, weight management, and hemoglobin A1c levels than using either medication alone, particularly for patients with metabolic or cardiovascular conditions [44,45].

Given that hyperlipidemia is a common comorbidity in T2DM, many patients are concurrently treated with statins, which are associated with a risk of liver dysfunction. In this context, the hepatoprotective properties of GLP-1R agonists may offer an additional therapeutic advantage by potentially mitigating statin-associated liver injury. This dual benefit positions GLP-1R agonists as a safer and more comprehensive treatment strategy for high-risk populations with T2DM and hyperlipidemia. Notably, treatment with LIRA, a GLP-1R agonist, alleviated oxidative stress, suppressed inflammatory response, and restored the imbalance between autophagy and apoptosis. These effects contributed to a reduction in elevated liver enzyme levels and the amelioration of histopathological alterations associated with ATO-induced liver dysfunction.

Despite these promising findings, the study has several limitations. Most importantly, further research is needed to evaluate the long-term efficacy and safety of LIRA in preventing or mitigating statin-induced liver dysfunction. Moreover, additional investigations are warranted to explore its potential as an alternative or adjunctive therapy for hepatoprotective effects in clinical settings. As such, the clinical utility of LIRA for managing ATO-induced liver dysfunction remains to be fully established.

Finally, it is important to acknowledge that this study represents a preclinical investigation conducted in a rat model, and while the findings are promising, they require further validation through clinical studies to confirm their relevance to human populations. Despite this, the translational potential of the current work is strengthened by the fact that both atorvastatin and liraglutide were administered through clinically relevant routes—atorvastatin orally and liraglutide via subcutaneous injection in humans, which closely parallels the oral and parenteral (intraperitoneal) administration used in this animal model. Therefore, the pharmacological context remains consistent, supporting the credibility of the proposed mechanisms. Nonetheless, future clinical study studies or observations should explore long-term safety, dose extrapolation, and clinical applicability in patient settings.

## Figures and Tables

**Table 1:** Primer sequences for mRNA expression.

Gene	Accession Number	Primer Sequence
HGMB-1	NM_012963.3	F: 5′-AGGCTG ACAAGGCTCGTTATG-3′ R: 5′-TGTCATCCGCAGCAG TGTTG-3′
TLR-4	NC_005104.4	F: 5′-GCTTGAATCCCTGCATAGAGG-3′ R: 5′-TGTCTCCACAGCCACCAGATTCTC-3
RAGE	NM_053336.2	F: 5′-CTGCCTCTGAACTCACAGCCAATG-3′ R: 5′-GTGCCTCCTGGTCTCCTCCTTC-3′
GLP1-R	NC_086038.1	F: 5′-GACCTGCCCTTGGAACCTCA-3′ R: 5′-AATGGCGGCACTCCAGATG-3′
β-Actin	NM_031144.3	F: 5′-TATCCTGGCCTCACTGTCCA-3′ R: 5′-AACGCAGCTCAGTAACAGTC-3′

## Data Availability

The datasets generated during and/or analyzed during the current study are available from the corresponding author upon request.

## List of Abbreviations

Akt	protein kinase B;
ALT	alanine aminotransferase;
AMPK	AMP activated protein kinase;
AST	aspartate aminotransferase;
ATO	atorvastatin;
GLP-1	glucagon like peptide-1;
HMGB-1	high-mobility group box-1;
IL-4	interleukin-4;
MAPK	mitogen activated protein kinase;
NADPH	nicotinamide adenine dinucleotide phosphate oxidase;
NFκB	nuclear factor kappa-B;
Nrf 2	Nuclear factor erythroid 2-related factor 2;
SOD	superoxide dismutase;
TLR-4	toll like receptor 4;
TNF-α	tumor necrosis factor alpha
